# Temporal changes within mechanical dyssynchrony and rotational mechanics in Takotsubo syndrome: A cardiovascular magnetic resonance imaging study

**DOI:** 10.1016/j.ijcard.2018.04.088

**Published:** 2018-12-15

**Authors:** Sören J. Backhaus, Thomas Stiermaier, Torben Lange, Amedeo Chiribiri, Pablo Lamata, Johannes Uhlig, Johannes T. Kowallick, Uwe Raaz, Adriana Villa, Joachim Lotz, Gerd Hasenfuß, Holger Thiele, Ingo Eitel, Andreas Schuster

**Affiliations:** a University Medical Center Göttingen, Department of Cardiology and Pneumology, Georg-August University, Göttingen Germany and German Center for Cardiovascular Research (DZHK), partner site Göttingen, Göttingen, Germany; b University Heart Center Lübeck, Medical Clinic II (Cardiology/Angiology/Intensive Care Medicine), University Hospital Schleswig-Holstein, Lübeck, Germany and German Center for Cardiovascular Research (DZHK), partner site Hamburg/Kiel/Lübeck, Lübeck, Germany; c Division of Imaging Sciences and Biomedical Engineering, King's College London, London, UK; d University Medical Center Göttingen, Institute for Diagnostic and Interventional Radiology, Georg-August University, Göttingen Germany and German Center for Cardiovascular Research (DZHK), partner site Göttingen, Göttingen, Germany; e Heart Center Leipzig, University of Leipzig, Department of Internal Medicine/Cardiology, Leipzig, Germany; f Department of Cardiology, Royal North Shore Hospital, The Kolling Institute, Northern Clinical School, University of Sydney, Sydney, Australia

**Keywords:** CMR, cardiovascular magnetic resonance, FT, feature tracking, URE, uniformity ratio estimate (dyssynchrony), CURE, circumferential URE, RURE, radial URE

## Abstract

**Background:**

The pathophysiological significance of dyssynchrony and rotation in Takotsubo syndrome (TTS) is unknown. We aimed to define the influence of cardiovascular magnetic resonance feature tracking (CMR-FT) dyssynchrony and rotational mechanics in acute and during clinical course of TTS.

**Methods:**

This multicenter study included 152 TTS patients undergoing CMR (mean 3 days after symptom onset). Apical, midventricular and basal short axis views were analysed in a core-laboratory. Systolic torsion, diastolic recoil and dyssynchrony expressed as circumferential and radial uniformity ratio estimates (CURE and RURE: 0 to 1; 1 = perfect synchrony) were compared to a matched control group (n = 21). Follow-up CMR (n = 20 patients; mean 62 days, SD 7.2) and general follow-up (n = 136; mean 3.3 years, SD 2.4) were performed.

**Results:**

CURE was initially reduced compared to controls (p = 0.001) and recovered at follow-up (p < 0.001) as opposed to RURE (p = 0.116 and p = 0.179). CURE and RURE discriminated between ballooning patterns (p = 0.001 and p = 0.045). Recoil was generally impaired during the acute phase (p = 0.015), torsion only in highly dyssynchronous patients (p = 0.024). Diabetes (p = 0.007), physical triggers (p = 0.013) and malignancies (p = 0.001) predicted mortality. The latter showed a distinct association with impaired torsion (p = 0.042) and dyssynchrony (p = 0.047). Physical triggers and malignancies were related to biventricular impairment (p = 0.004 and p = 0.026), showing higher dyssynchrony (p < 0.01), greater reduction of left ventricular function (p < 0.001) and a strong trend towards increased mortality (p = 0.074).

**Conclusion:**

Transient circumferential dyssynchrony and impaired rotational mechanics are distinct features of TTS with different severities according to the pattern of ballooning. Patients with malignancies and precipitating physical triggers frequently show biventricular affection, greater dyssynchrony and high mortality risk.

## Highlights

• Transient circumferential dyssynchrony is a distinct feature of acute TTS.
• Recoil is impaired in the acute phase, whilst torsion remains preserved.
• High amounts of dyssynchrony are associated with additionally impaired torsion.
• Precipitating physical triggers are related to biventricular impairment.

## 1 Introduction

Takotsubo syndrome (TTS), also referred to as Takotsubo cardiomyopathy, left ventricular apical ballooning syndrome and stress-induced cardiomyopathy [1], is an increasingly recognized heart condition which often occurs related to a stressful trigger with an acute onset and symptoms comparable to those of an acute coronary syndrome [2].

Myocardial dysfunction is usually not related to the typical supply territories of coronary arteries [1,2]. Left ventricular (LV) [3], left atrial (LA) [4] and right ventricular (RV) [5] dysfunction have been shown in TTS. Ventricular impairments include hypokinesia, akinesia or dyskinesia in a peculiar pattern within the apical, midventricular and basal segments, although the apical ballooning pattern is the most common and typical entity. Cardiovascular magnetic resonance (CMR) plays an important role in the assessment, diagnosis confirmation and differential diagnosis of TTS. The exact underlying pathophysiology of TTS remains unknown [4] and since recent data suggest an underlying ischemic aetiology [6] it has been included within the definition of myocardial infarction with non obstructive coronary arteries (MINOCA) [7]. Despite the frequent reversibility of the initial myocardial dysfunction, mounting evidence suggests diminished short and long-term prognosis [[8], [9], [10]]. Studies tried to establish specific laboratory testing to guide clinical management [11], however specific biomarkers for effective prognostic patient evaluation are still lacking.

CMR represents the reference standard for cardiac morphology, function and tissue characterisation with strong diagnostic value in TTS. Furthermore, the impact of LV [12] and RV involvement [5] on adverse events in TTS has already been shown. Recently, CMR feature tracking (CMR-FT), an analogue to speckle tracking echocardiography (STE), has been introduced as a novel method for quantitative evaluation of cardiovascular function [13]. In addition to robust and reproducible assessments of biventricular systolic myocardial function [13,14] novel CMR-FT applications allow complex quantification of myocardial mechanics such as myocardial twist or torsion and dyssynchrony [[15], [16], [17], [18]].

Previous studies based on 2D- [19] and 3D [20]-STE strain assessment suggested a role of both impaired ventricular twist [21] and ventricular dyssynchrony within the acute phase of TTS [20]. Little is known about neither their pathophysiological significance nor their prognostic implications in TTS. Hence, the aim of the present study was to assess the impact of CMR-FT derived LV rotational mechanics and dyssynchrony parameters as novel diagnostic and prognostic tools in the evaluation of TTS.

## 2 Methods

### 2.1 Study population

This international multicenter study included 152 patients (Table 1) at the University Hospitals of Leipzig - Heart Center (n = 125) and Goettingen (n = 2), Germany and at King's College London - St. Thomas' Hospital (n = 25), United Kingdom. All participants presented with symptoms of acute chest pain and dyspnoea. Patients were diagnosed with TTS on the basis of the Mayo Clinic criteria [1] consisting of transient LV-segmental hypokinesia, akinesia or dyskinesia beyond a single epicardial vascular distribution territory, absence of coronary artery disease associated with the area of the wall movement disorder, new electrocardiographic abnormalities (e.g. ST-elevation or T-wave deformation) and absence of pheochromocytoma and myocarditis. Patients were retrospectively selected if these criteria were fulfilled and CMR had been conducted during the clinical routine. A stressful trigger could be present, although was not required for final diagnosis. All patients included underwent CMR scanning within the acute onset of TTS (mean of 3 days, SD 2,3). Whilst every patient received a follow-up assessment of LV function within 6 months of initial consultation by transthoracic echocardiography, follow-up CMR data was available for a subgroup of 20 patients. Furthermore, long-term survival follow-up data were obtained by telephone contact with either the patients, their relatives or treating physicians. All adverse events were checked upon in documented medical records. CMR-FT analyses were performed retrospectively in b-SSFP CMR cine images from consecutively and prospectively enrolled TTS patients who underwent imaging during their clinical routine management.

**Table 1:** Patients demographic data.

Parameter	TTS patients	Control group	p
Population n	152	21	
Gender F/M	127/25	16/5	0.405
Age	69 ± 11.1	62.1 ± 17.4	0.125
LVEF (%)	48 ± 9	67.6 ± 4	<0.001
RURE	0.74 ± 0.1	0.77 ± 0.08	0.116
CURE	0.81 ± 0.1	0.9 ± 0.05	<0.001
Diastolic recoil (° cm^−1^)	−2.5 ± 1.54	−3.34 ± 1.6	0.015
Systolic torsion (° cm^−1^)	2.46 ± 1.53	2.34 ± 1.32	0.901

The table shows the different characteristics of TTS patients in the acute phase and a healthy control group. Values expressed as mean ± standard deviation. The Mann-Whitney U test was used to determine significant differences between TTS patients and the control group. LVEF: left ventricular ejection fraction, RURE: radial - CURE: circumferential uniformity ratio estimate.

### 2.2 Control group

The control group consisted of 21 patients at the University Hospital Goettingen (16 female, 5 male, mean age 70.8 SD 17.4 years) who underwent CMR scanning within the clinical routine and were found to have normal cardiovascular morphology and unimpaired biventricular function.

### 2.3 CMR imaging

All study participants underwent a standardised CMR imaging protocol on a 1.5 or 3.0 Tesla magnetic resonance scanner (Achieva and Ingenia, Philips Medical Systems, Best, The Netherlands as well as Skyra and Symphony, Siemens Healthcare, Munich, Germany) as previously described [3]. In brief, the CMR protocol included ECG-gated short axis balanced steady state free precession (SSFP) images for the assessment of ventricular function as well as regional wall motion abnormalities, maximum segmental wall thickness was measured in the short axis orientation. Late gadolinium enhancement imaging was performed 10–15 min after contrast application for fibrosis and necrosis evaluation.

### 2.4 CMR feature tracking

CMR-FT was performed as previously described [17]. Offline image analysis was performed with the certified CMR evaluation software 2D CPA MR, Cardiac Performance Analysis, Version 1.1.2, provided by TomTec Imaging Systems, Unterschleissheim, Germany. Experienced investigators at the core laboratory at the University Medical Centre Goettingen with proven low inter- and intraobserver variability and high reproducibility [16,18,22] analysed the data from all participating study sites. The software is validated and has been used in several prior studies [13,14,23]. Scans with insufficient image quality were excluded. The borders were tracked in the short axis planes at basal, midventricular and apical locations as previously described [17,18]. In brief, endocardial and epicardial borders were manually traced using a point-and-click approach at end-diastole in ECG-gated SSFP images. By tracking 48 voxels the software consecutively renders automatically endo- and epicardial borders in the analysed slices. Tracking accuracy was visually reviewed, where needed adjustments were made to the initial manually traced border and the tracking cycle was repeated. All analyses consisted of 3 measurement repetitions with subsequent averaging [23]. SA slices were selected based on standard operating procedures in the core laboratory. Briefly, the most apical slice showing endsystolic intracardiac blood volume and the most basal slice without appearance of the outflow tract throughout the cardiac cycle were selected for further analyses. The midventricular slice is selected in similar distance of apical and basal slices and showed presence of the papillary muscles.

### 2.5 Assessment of myocardial function

**Fig. 1:**
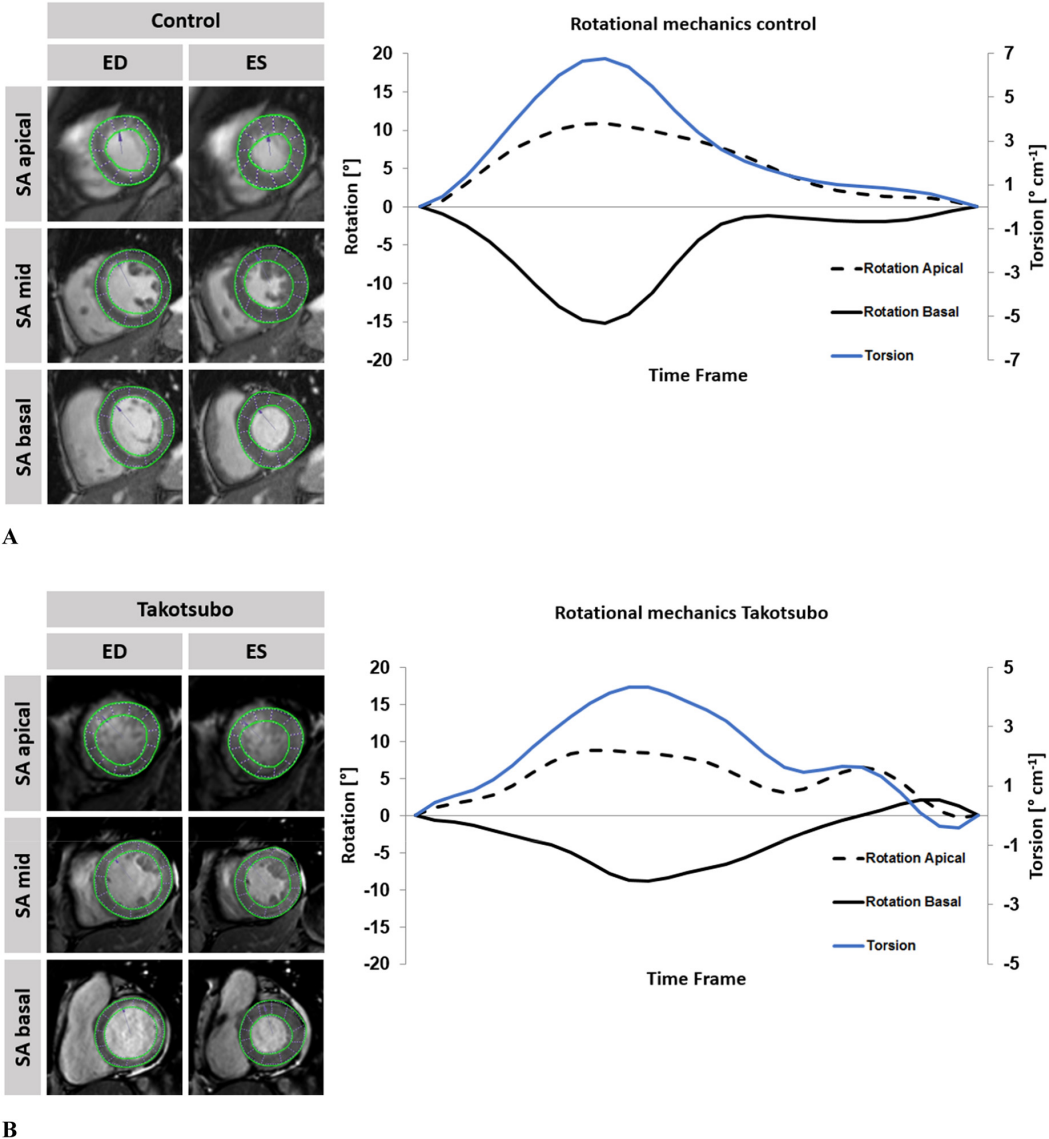
A Apical, midventricular and basal short axis (SA) views of the left ventricle in a representative patient with normal biventricular function. On the left, enddiastolicly (ED) and endsystolicly (ES) tracked endo- and epicardial borders are being displayed in apical, midventricular and basal short axis views of a representative patient with normal biventricular function. On the right respective rotation and torsion is being plotted over a single cardiac cycle. B Apical, midventricular and basal short axis (SA) views of the left ventricle in a representative TTS patient with apical ballooning. On the left, enddiastolicly (ED) and endsystolicly (ES) tracked endo- and epicardial borders are being displayed in apical, midventricular and basal short axis views of a representative patient with apical ballooning Takotsubo syndrome. On the right respective rotation and torsion is being plotted over a single cardiac cycle.

Systolic and diastolic torsion (twist per length) were defined and calculated as the difference in counter-clockwise apical (ϕ_apex_) and clockwise basal rotation (ϕ_base_) divided by the distance (D) between the imaging planes [17,18]. The rotation was calculated from the angular displacement of the tracked voxels. Averaged data from the 3 independent measurements were used to calculate endocardial systolic torsion and diastolic recoil. Rotation was plotted against the time frame of the cardiac cycle. The difference of apical and basal rotation in each time frame was divided by their slice distance to obtain torsion correcting for cardiac geometry such as variable LV lengths [17] (Fig. 1A & B).

$$T=\frac{\phi_{\mathrm{apex}}-\phi_{\mathrm{base}}}{D}\;\left[{}^{\circ}\;cm^{-1}\right]$$

### 2.6 Uniformity ratio estimates

Assuming equal strain across the myocardium in a perfectly synchronous heart at a given time-point, strain consequently changes along the myocardial sections at a given point in time in a dyssynchronous heart. Circumferential and radial strains were plotted against spatial positions and resulting oscillations within the plots represented myocardial dyssynchrony [24]. Fourier analysis of the corresponding plots and averaging in time and space resulted in circumferential and radial uniformity ratio estimates (CURE and RURE) as laid out elsewhere [24]. Resulting values range between 0 and 1 representing complete dyssynchrony and perfect synchrony, respectively [15].

### 2.7 Statistical analysis

Continuous parameters are presented as mean and standard deviation (SD) as measure of dispersion. Categorical parameters are given as absolute numbers and percent values. The assumption of normally distributed continuous variables was tested using the Shapiro-Wilk test. The Wilcoxon signed-rank test was used to evaluate continuous parameters in dependent samples, and the Mann-Whitney-U test for independent samples. Differences for categorial variables were tested using the chi-squared test or if applicable 2-tailed Fisher exact test. In a first step, comparisons were made between patients with TTS and control subjects. In a second step, TTS patients were classified and compared according to their extent of dyssynchrony (greater or less than the median). Correlation between continuous parameters was assessed using the spearman's rank correlation coefficient ranging from rho = −1 to rho = 1. Both rho = −1 and rho = 1 indicated a perfect correlation, whereas rho = 0 indicated no correlation [25]. For mortality risk evaluation, Cox proportional hazard models were implemented. An alpha level of 0.05 was chosen for statistical significance. All p-values provided are two-sided. Statistical calculations were performed using IBM SPSS Statistic Software Version 24 for Windows (IBM, Armonk, NY, USA). Due to the small sample size, the basal ballooning type (n = 2) was not considered for statistical comparisons.

## 3 Results

### 3.1 TTS study population

The cohort showed representative demographics and characteristics for TTS (Table 1) [10]: 83.6% of patients were female, the mean age was 69 years (SD 11.1) and 60.5% of the cases reported a stressful event before acute onset of symptoms (56.5% physical, 43.5% emotional). Initial CMR was performed in the acute phase of TTS (in mean within 3 days of hospitalization, SD 2.2). Due to severe CMR artefacts (e.g. breathing motion) with resulting insufficient tracking accuracy recoil (n = 18), torsion (n = 14) and dyssynchrony (n = 8) parameters could not be obtained in all cases. The ballooning patterns were distributed as follows: 108 patients presented with apical ballooning (71.1%), 40 (26.3%) with midventricular and 2 (1.3%) presented with basal ballooning (Table 2). The remaining 2 patients (1.3%) did not show classical ballooning with either clear apical, midventricular or basal patterns. Patients with apical ballooning were older than patients with midventricular ballooning (p = 0.02). The was no presence of late gadolinium enhancement (LGE) in any patient as defined by >5 SD compared to remote myocardium. Pericardial effusion was seen in 94 of 117 (80.3%) patients and myocardial oedema in 47 of 125 (37.6%) patients. Maximal wall thickness was in mean 9.5 mm (SD 1.4). Mean creatine kinase concentration [4 μmol/l*s (SD 5.2)] was close to the upper limit of the normal range. Disturbances of repolarization including 37.5% ST-elevation, 4.2% ST-depression and 51.7% T-wave inversion were present during the acute phase of TTS [10], however without association to all-cause mortality. Follow-up revealed a mortality of 11.2%. Out of these 17 patients 8 died due to non-cardiovascular, 4 due to cardiovascular causes and 5 remained unidentified. Cardiovascular mortality included shock, pulmonary embolism and arrhythmia. 16 patients (12.8%) had a malignancy in their history significantly associated with mortality (p = 0.001), however only directly causing death in 5 patients. Malignancies were significantly more often pronounced in male patients (p = 0.007). Neither hypertension (p = 0.44), nor hyperlipidaemia (p = 0.44), nicotine (*p* = 0.46) and an impaired LVEF below 50% (p = 0.131) were associated with increased mortality. Diabetes was the only cardiovascular risk factor showing an association with increased mortality (p = 0.007). A stressful trigger in general was not associated with increased mortality (p = 0.127), though a physical trigger was (p = 0.013). Male sex was associated with increased risk for mortality (p = 0.024). Except for hyperlipidaemia (p = 0.038), there were no differences in cardiovascular risk factors and trigger types between the genders (p = 0.340 to 0.799). The 20 follow-up patients did not differ from the whole cohort in terms of general characteristics, cardiovascular risk factors, medication and parameters of ventricular function (p > 0.05 for all parameters).

**Table 2:** Overview for ballooning patterns and associated ventricular function parameters.

Ballooning pattern	Apical (n = 108)	Midventricular (n = 40)	Basal (n = 2)	p
LVEF (%)	43.5 ± 7.9	50.9 ± 8.4	53.0	<0.001
RURE	0.73 ± 0.1	0.77 ± 0.08	0.60	0.045
CURE	0.79 ± 0.1	0.85 ± 0.08	0.76	0.001
Diastolic recoil (° cm^−1^)	−2.43 ± 1.45	−2.45 ± 1.47	−3.43	0.84
Systolic torsion (° cm^−1^)	2.28 ± 1.37	2.88 ± 1.79	2.31	0.137

The table shows the different ballooning patterns and their associated ventricular function parameters in TTS patients. Values expressed as mean. The Mann-Whitney U test was used to determine significant differences between apical and mid-ventricular ballooning patterns, basal patterns were excluded in the statistical analysis. LVEF: left ventricular ejection fraction, RURE: radial - CURE: circumferential uniformity ratio estimate.

### 3.2 Uniformity ratio estimates

RURE (p = 0.045) and CURE (p = 0.001) discriminated between apical and midventricular ballooning patterns. Patients with apical ballooning were more associated with dyssynchrony than those with midventricular ballooning. Whilst patients with myocardial oedema showed higher circumferential (p = 0.043) and a trend towards higher radial dyssynchrony (p = 0.074), RURE (p = 0.045) and CURE (p = 0.001) continuously discriminated between ballooning patterns in the absence of myocardial oedema. Contrary, pericardial effusion was associated with lower radial dyssynchrony (p = 0.041). There were no gender specific differences in dyssynchrony (CURE p = 0.731, RURE p = 0.898). Myocardial hypertrophy did not correlate with CURE (rho = 0.04, p = 0.642) and RURE (rho = 0.021, p = 0.804). Patients with malignancies suffered from higher dyssynchrony (CURE p = 0.044, RURE p = 0.052).

CURE was impaired in acute TTS compared to control subjects (p < 0.001) and recovered at follow-up (p = 0.001). There was no significant difference in RURE between TTS patients and the control group during the acute onset as well as between the acute phase and follow-up (p = 0.116 and p = 0.179, respectively).

Lower amounts of dyssynchrony were associated with higher LVEF (CURE Rho = 0.47, p < 0.001 and RURE Rho = 0.34, p < 0.001). Furthermore, there was an association of CURE with systolic torsion (Rho = 0.192, p = 0.029) and diastolic recoil (Rho = 0.175 p = 0.047). There was no association of dyssynchrony estimates and mortality.

### 3.3 Torsion

Rotational mechanics including recoil and torsion were not associated with pericardial effusion and myocardial oedema (p = 0.217 to 0.6) and did not correlate with myocardial hypertrophy (rho = −0.16 to 0.06, p = 0.457 to 0.852, respectively). Cardiovascular risk factors did not affect recoil and torsion, including hypertension (p = 0.299 and p = 0.285), diabetes (p = 0.198 and p = 0.362), hyperlipoproteinemia (p = 0.930 and p = 0.592) and nicotine use (p = 0.444 and p = 0.975). Diastolic recoil was higher in man compared to woman during the acute phase of TTS (p = 0.033), whereas systolic torsion did not differ (p = 0.312). Whilst systolic torsion remained preserved in the acute phase with no significant differences in comparison to the control group (p = 0.901), diastolic recoil was significantly impaired (p = 0.015) (Table 1). Both parameters remained unchanged at follow-up (p = 0.896 and p = 0.52, respectively). However, there was a numerical increase in diastolic recoil at follow up (−2.6 to −2.75°cm^−1^) with a reduction of the initial difference in diastolic recoil compared to the control group (control −3.34 SD 1.58°cm-1 compared to follow-up −2.74 SD 1.33 cm-1, p = 0.232). After grouping the patients according to the median of CURE, significantly lower values of systolic torsion (mean 2,18 SD 1.38 cm^−1^ compared to mean 2.76 SD 1.64°cm^−1^, p = 0.024) and a trend towards lower values of diastolic recoil (mean − 2.27 SD 1,43° cm^−1^ compared to mean − 2.74 SD 1.62° cm^−1^, p = 0.051) were found in the group with a higher degree of dyssynchrony. Patients with malignancies suffered from significantly impaired systolic torsion (p = 0.046) as well.

### 3.4 Biventricular ballooning

Patients with biventricular affection were significantly older (p = 0.001) and had more stressful events in their history (p = 0.002) as compared to the rest of the study group. However, distinguishing physical and emotional triggers, the former emerged as relevant (p = 0.004) for biventricular affection whereas the latter did not (p = 0.61). Additional RV-involvement presented more often in patients with malignancies (p = 0.024). Biventricular affection was associated with significantly higher circumferential dyssynchrony (p < 0.001) and impaired LVEF (p < 0.001) with a trend towards higher mortality (p = 0.074).

## 4 Discussion

The current work bears several notable findings based on the investigation of CMR-FT derived dyssynchrony and rotational performance assessments in a relatively large TTS multicentre study collective.

First, transient dyssynchrony is a distinct feature of TTS with higher amounts in the acute setting that resolve over time in parallel with restoration of overall systolic function. Second, TTS patients show overall impairment of diastolic recoil whilst systolic torsion remains preserved. Third, in patients with high amounts of dyssynchrony additional impairment of systolic torsion occurs. Fourth, pre-existing malignancies and precipitating physical triggers are related to biventricular impairment and associated with impaired myocardial mechanics. Finally, these novel parameters can be easily assessed using CMR-FT and may have a role in pathophysiology assessment in TTS.

### 4.1 Dyssynchrony

The current data demonstrate the presence of distinct myocardial dyssynchrony in the acute phase of TTS. Whilst CURE shows transient impairment, both CURE and RURE show close associations with the different ballooning patterns and associations with other cardiac performance parameters. One would assume that ballooning patterns influence cardiac fibres and therefore the mechanics of ventricular contractility. In this study, this is predominantly reflected in the (dys-)synchrony parameters URE that accurately discriminate between different ballooning patterns with the highest dyssynchrony associated with apical ballooning. Additionally, dyssynchrony is accelerated by transient myocardial oedema, which has been previously shown in acute myocardial infarction [26]. However, cardiovascular risk factors or myocardial hypertrophy are not associated with dyssynchrony, suggesting a distinct role in TTS rather than association with pre-existing diseases. Importantly the follow-up data reveal that resynchronisation represents a part of the recovery, which can be appreciated from increased CURE values. Despite dyssynchrony resolution, EF and strain impairment have also been demonstrated by echocardiography [27] and CMR [28] and represent important aspects of LV recovery [28] in TTS. This observation is also paralleled by the correlation of URE and EF and in accordance with previously reported observations in chronic heart failure [29]. Although CURE and RURE have been demonstrated to equally differentiate between healthy controls and non-ischemic cardiomyopathy subjects [15], within the current study, CURE had higher correlation values with LVEF and was more affected within the acute phase as compared to RURE. Whether this reflects pathophysiological differences with more circumferential dyssynchrony as compared to radial dyssynchrony in acute TTS remains speculative. Notwithstanding these considerations, Helm and colleagues proposed superiority of circumferential dyssynchrony evaluation over radial assessments [30]. Furthermore, radial strain assessed by CMR-FT has been shown to have lower intra- and inter-observer reproducibility [31], although Onishi et al. [32] demonstrated reasonable agreement for CMR-FT measurements of radial dyssynchrony and STE. Historically, the aetiology of TTS has been explained by an independent cardiomyopathy, however, the high prevalence of cardiovascular risk factors has recently triggered the hypothesis of underlying acute myocardial ischaemia [6,33,34]. Regional microvascular blood flow misdistribution potentially reinforced by endothelial dysfunction [35] may trigger regional wall motion differences and thus dyssynchrony. Indeed, risk factors for coronary artery disease are largely present amongst TTS patients in the present collective and consequently it is interesting to speculate whether a vascular aetiology may account for some of the current findings [6].

### 4.2 Rotational mechanics

There is a link between the amount of dyssynchrony and rotational performance as expressed by LV-torsion parameters which have also been assessed within this study. Both systolic torsion and diastolic recoil show a relatively weak but statistically significant correlation with CURE. Interestingly, active systolic torsion was preserved in the overall collective and did not differentiate between ballooning patterns, whilst a general impairment of passive diastolic recoil was observed during the acute phase of TTS. Furthermore, patients with high amounts of dyssynchrony as defined by the median of CURE developed significantly lower active systolic torsion as opposed to patients with less dyssynchrony. In this setting myocardial impairment due to myocardial ballooning with underlying microvascular dysfunction and activation of the ischaemic cascade first affects passive recoil, prior to also influencing active systolic torsion. Rotational mechanics have indeed shown to be influenced by regional differences in myocardial fibre impairment for example after ischemia [36]. Such regional fibre impairments due to microvascular ischemia and ballooning [17] would supposedly also be present in TTS [6,34]. It is important to note that this observation seems to be a distinct feature in the pathophysiology of TTS since there is no association with risk factors or hypertrophy.

### 4.3 Clinical considerations and prognostic implications

Until recently regarded as transient and benign, studies demonstrated a pronounced increase in short and long-term mortality TTS [10,37,38]. The pathophysiological properties underlying this increased risk for adverse events have yet to be elucidated. LVEF is a clinically well-established global parameter in the assessment of cardiac function, but it fails to detect regional functional impairments. However, similar to LVEF, the cardiovascular risk factors hypertension, hyperlipidaemia and nicotine were not associated with increased mortality, exclusively diabetes did show an association. In contrast, prevailing evidence reported a low prevalence of diabetes amongst TTS patients, with protective effects due to diabetic autonomic neuropathy and thus amelioration of catecholamine-mediated effects [[39], [40], [41]] which are said to impact TTS [38,42]. Notwithstanding, to date there are no prospective trials evaluating the therapeutic management of TTS including the use of Beta blockers with regards to catecholamine-mediated effects nor regarding affection of clinical course or outcome [10]. In this context, the prevalence of 28.2% of patients suffering from diabetes in the current study collective was relatively high. Nonetheless and in line with our findings, recent reports from the international multi-centre registry GEIST indicate a substantially higher incidence of diabetes as well as associated worsening of outcome [43]. Besides diabetes, malignancies have been reported to influence mortality in TTS [37,44]. With a prevalence of 12.5%, the presence of malignancies was lower compared to the literature [37,44]. Although there was a significant association of malignancy and mortality, only 5 deaths were directly attributable to cancer. In this context, inflammatory and neurohormonal mechanisms are discussed to increase cardiac death and overall mortality [44]. Whilst malignancies are clearly associated with long-term survival probability, these processes seem to impact the acute phase as well. Patients suffering from malignancies showed distinctly more often biventricular impairment, including higher dyssynchrony and impaired systolic torsion. Furthermore, the presence of a precipitating physical (and not emotional) trigger resulted in higher mortality which may be attributed by more frequent biventricular impairment associated with higher dyssynchrony and reduced LVEF [45]. Biventricular affection itself has previously been shown to be associated with increased in-hospital as well as long-term mortality [5]. Interestingly, in the current study additional RV involvement was associated with higher dyssynchrony as well as lower LVEF but only a trend towards increased mortality.

Male sex was associated with higher mortality as previously reported [10]. Whilst only hyperlipidaemia showed a gender difference amongst the cardiovascular risk factors with higher prevalence in male patients, the only univariate significant parameter diabetes was distributed similarly between the genders. A physical stressor, which has shown associations with increased mortality, was distributed similarly as well. Hence, the elevated risk attributed to male sex seems to be irrespective of stressor type or cardiovascular risk factors. A mild increase in diastolic recoil accompanied by equally high torsion and dyssynchrony parameters in men is unlikely the cause of mortality differences. Interestingly, malignancies were significantly more common amongst male patients, thus explaining the differences in survival probability, that was observed in the present study population.

### 4.4 Clinical challenges

There is a clear need to establish novel diagnostic imaging parameters to assess and quantify TTS associated short and long-term risks for adverse clinical events. Whilst we could demonstrate a clear relationship between the onset of TTS and both dyssynchrony as well as rotational mechanics, we could not provide incremental diagnostic value in risk stratification. Although we provide the to date largest TTS collective undergoing CMR diagnostics for detailed evaluation of myocardial rotational mechanics and dyssynchrony, more clinical endpoints hence higher patient numbers will be needed to statistically reliable define the impact of transiently impaired ventricular mechanics on in-hospital mortality as well as long-term outcome. Nevertheless, we could demonstrate distinct changes during the acute phase of TTS and at follow-up, thus providing insights into their importance in TTS. This adds to the existing body of evidence showing the influence of CMR-FT derived myocardial strain assessment such as dilated cardiomyopathy [46] and ischemic heart disease [47]. CMR-FT parameters have been examined and validated in multiple prior studies, and did show good intra- and interobserver as well as interstudy reproducibility [[16], [17], [18],^22^]. Since CMR-FT can be conducted in conventional SSFP cine images acquired during routine CMR scanning, the additional assessment of (dys-) synchrony and rotational parameters by the means of CMR-FT seems reasonable according to the current data and could be of significant utility for improved understanding of TTS, which pathophysiology remains to be fully elucidated [48,49].

### 4.5 Study limitations

Some data had to be excluded from further analysis due to insufficient image quality, e.g. because of breathing motion, resulting in insufficient tracking performance. Whether a selection bias took place due to worse image quality and tracking in relatively sicker patients remains elusive. Generally, the inclusion of relatively stable patients that are able to undergo CMR scanning may have resulted in a selection bias leading to a lower number of events in our study collective [9]. CMR follow-up data were available for 20 patients only, which may not necessarily reflect the whole study cohort, however there were no statistical differences between patients with and without follow-up (data not shown). Although, the present study is the largest CMR study in TTS to date involving detailed myocardial deformation, further studies with more patients and thus events during follow-up are needed for adequately powered conclusions regarding cardiovascular mortality. Since the cause of death could not definitely be confirmed in 5 cases statistical analysis was conducted for overall mortality only. Whilst LVEF is a known predictor of outcome in TTS, this association could not be shown in our study collective. This might be due to the time between hospitalization and referral to MRI as well as acute complications in the acute phase of TTS such as shock or pulmonary oedema prohibiting patients from being scanned and consequently in a selection of healthier patients. The control group did not consist of healthy volunteers but of patients that were referred for routine CMR scanning. However, these age and sex matched patients were found to have normal biventricular function as well as regular ventricular strain parameters in line with a recently published normal values for healthy subjects [50].

## 5 Conclusion

This study underlines the presence of both dyssynchrony and impaired ventricular rotational parameters in TTS patients. Essentially, dyssynchrony is increased within the acute phase of TTS, recovers over time and is most pronounced in apical ballooning. Dyssynchrony further affects rotational mechanics with reduced rotational performance in patients with high amounts of dyssynchrony. Patients with malignancies and precipitating physical triggers frequently show biventricular affection, greater dyssynchrony, reduced left ventricular ejection fraction and subsequent high mortality. CMR-FT offers reliable and accurate assessments of (dys-) synchrony as well as rotational parameters that may be of significant utility for improved pathophysiological evaluation of TTS.

## Disclosures

None.

## Funding sources

This study was supported by a German Center for Cardiovascular Research (DZHK) research grant.

## Declarations

### Ethics approval and consent to participate

The study was approved by the local ethics committees at the University Hospitals of Luebeck, Goettingen and London, St. Thomas Hospital. The study was conducted according to the principles of the Helsinki Declaration. All patients provided written informed consent before participation.

## Competing interests

The authors declare that they have no competing interests.

## Funding

DZHK (81X2700114) - Deutsches Zentrum für Herz-Kreislauf-Forschung eV (German Center for Cardiovascular Research). PL held a Sir Henry Dale Fellowship jointly funded by the Wellcome Trust and the Royal Society (grant n.099973/Z/12/Z), and holds a Wellcome Trust Senior Research Fellowship (grant n.209450/Z/17/Z).

## Authors' contributions

SB, TS, IE and AS designed the study protocol, performed data acquisition, performed statistical analyses and drafted the manuscript. TL and PL performed data acquisition/analysis and revised the manuscript. JU performed statistical analyses. JK, UR, AC, AV, JL, GH and HT revised the manuscript and participated in the scientific discussion during the study. All authors read and approved the final manuscript.
